# Effects of Phenobarbital and Prednisolone on Neurological Signs of Canine Distemper

**DOI:** 10.1002/vms3.70479

**Published:** 2025-08-20

**Authors:** Ali Asghar Sarchahi, Mohammad Arbabi, Hadi Mohebalian

**Affiliations:** ^1^ Department of Clinical Sciences Faculty of Veterinary Medicine Ferdowsi University of Mashhad Mashhad Iran; ^2^ Graduated from Faculty of Veterinary Medicine Ferdowsi University of Mashhad Mashhad Iran; ^3^ Department of Pathobiology Faculty of Veterinary Medicine Ferdowsi University of Mashhad Mashhad Iran

**Keywords:** canine distemper virus, dog, neurological distemper, phenobarbital, prednisolone

## Abstract

**Background:**

Canine distemper virus (CDV) is a highly infectious and often fatal disease in dogs, affecting various systems. Despite vaccination efforts, cases of distemper, especially the neurological form, remain a global concern due to its high fatality rate.

**Objective:**

To investigate the effectiveness of phenobarbital and prednisolone in treating the neurological form of canine distemper (CD).

**Methods:**

Thirty‐five dogs with neurological signs of CD were included in the study after careful clinical examination. Confirmation of CD was based on clinical signs, rapid diagnostic tests, and RT‐PCR testing of blood and/or cerebrospinal fluid (CSF). Dogs were treated with oral phenobarbital (2.5 mg/kg) and prednisolone (0.55 mg/kg) every 12 h. Treatment outcomes were categorised as recovered, died, or euthanised.

**Results:**

Out of the 35 dogs, 25 tested positive for CDV. Among positive cases (*n* = 25), two dogs mostly recovered, one dog partially recovered, one dog remained unchanged, 18 died (15 died naturally and three were euthanised), and three dogs were lost to follow‐up. In the negative test group (10 dogs), eight dogs died, the outcome of one dog was unknown and one dog remained unchanged. Disease duration ranged from 2 to 586 days (average: 72 days in positive cases, 59.2 days in negative cases). The low recovery rate (8%) suggests limited effectiveness of the treatments used, including prednisolone, particularly for myoclonus, which was the most frequent clinical sign. Regarding seizure management, while our study observed some effectiveness of phenobarbital in controlling seizures, it's important to note that the efficacy of phenobarbital can vary.

**Limitations:**

Small sample size and owner non‐compliance limited the study.

**Conclusions:**

Our findings suggest limited benefit from prednisolone for neurological CD. Further research is necessary to develop more effective treatment strategies for this devastating canine viral disease.

## Graphical abstract

This study investigates the effects of phenobarbital and prednisolone on neurological signs of canine distemper (CD) in 35 dogs. Among 25 dogs testing positive for canine distemper virus (CDV), treatment outcomes showed limited recovery with prednisolone (8% recovery rate) and effective seizure control with phenobarbital. In the negative group, 80% died or remained unchanged. The findings highlight the need for further research to develop more effective treatment strategies for this serious viral disease affecting dogs.

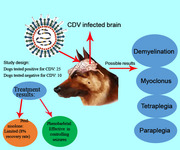

## 1 Introduction

Canine distemper virus (CDV), a member of the genus Morbillivirus within the Paramyxoviridae family, causes a highly contagious and often fatal disease in dogs worldwide (Beineke et al. 2009). Canine distemper (CD) is the second most deadly viral disease in dogs after rabies (Latha et al. 2007). Canine distemper virus is an enveloped, single‐stranded, negative‐sense RNA virus that encodes six structural (nucleocapsid N, fusion F, hemagglutinin H, matrix M, phospho‐P and large‐L proteins) and two nonstructural (C and V proteins) proteins similar to other paramyxoviruses (Carvalho et al. 2012). The virus affects various systems, such as respiratory, digestive, skin and nervous systems, presenting a wide array of clinical signs (Xue et al., 2019; Arbabi et al., 2022). The severity of clinical signs is influenced by factors including age, immune status, and the virulence of the CDV strain (Ranjithkumar and Dey 2021). While vaccination has demonstrably reduced distemper incidence, cases still occur worldwide, even in vaccinated animals (Feijóo et al. 2021; Sarchahi et al. 2022b). Recovery from respiratory and gastrointestinal signs is not uncommon (Arbabi et al. 2022), but neurological signs often show poor improvement (Sykes 2022). The neurological form of distemper manifests a wide variety of clinical signs, including seizures, vestibular and cerebellar signs, myoclonus, optic nerve involvement, and spinal cord deficits such as paraparesis, tetraparesis, and tetraplegia (Sarchahi et al. 2022b). These neurological signs are typically severe, progressive, and irreversible. Despite intensive treatment, the fatality rate of the disease remains high (Mousafarkhani et al. 2023), ranging from 30–80% in susceptible animals and up to 100% in ferrets (Wyllie et al. 2016). Current treatments involve supportive care, corticosteroids, and anticonvulsants; however, no definitive and effective treatment has been established yet (Sykes 2022). Treatments for the neurological form have shown limited success, leading to many dogs being euthanised due to a presumed poor outcome (Sykes 2022). Numerous studies have been conducted to find effective treatments for the neurological form of distemper (Teixeira et al. 2009; Bogdanchikova et al. 2016; Liu et al. 2016; Gastelum‐Leyva et al. 2022; Sarchahi et al. 2022b). While some studies have reported treatment success using various approaches (Teixeira et al. 2009; Sarchahi et al. 2022b), others have not shown significant progress (Bogdanchikova et al. 2016; Liu et al. 2016). While some texts suggest that corticosteroids and anticonvulsants may offer symptomatic relief in neurological distemper (Sykes 2022), their effectiveness lacks documented validation. Therefore, the aim of this study was to investigate the effect of phenobarbital and prednisolone on the neurological form of distemper. This study reported the long‐term treatment outcomes of phenobarbital and prednisolone in 35 dogs with the nervous form of distemper.

## 2 Materials and Methods

This study was conducted in accordance with the guidelines for the care and use of animals in research and was approved by the Ethics Committee of Ferdowsi University of Mashhad, Mashhad, Iran (Approval ID: IR.UM.REC.1399.120).

In this study, 35 dogs with neurological signs suggestive of canine distemper were enrolled. The inclusion criteria were based on the presence of specific neurological signs associated with distemper, such as myoclonus, paraparesis, tetraparesis/plegia, cerebellar (ataxia, dysmetria, intention tremors, nystagmus, decerebellate rigidity) and vestibular (ataxia, head tilt, tight circling falling, nystagmus and postural reaction deficits) signs, as well as focal and generalised seizures. A comprehensive clinical assessment was conducted on all dogs meeting these criteria. While the primary focus was on neurological manifestations, dogs exhibiting both neurological and systemic signs (digestive, respiratory, and dermatologic) were included in the study. However, to maintain the focus on neurological involvement and avoid potential confounding factors, we excluded dogs presenting solely with systemic signs without any neurological involvement. Additionally, animals with a history of phenobarbital administration or those that had previously received corticosteroid treatment were not included in the study. A written informed consent was obtained from all animal owners prior to study enrolment.

A detailed history was obtained for each enrolled dog, including age, sex, breed, housing type (indoor, yard, garden, and herd), feeding practices (commercial, homemade, and mixed food), vaccination history, and any prior medical conditions or surgeries (Tables 1 and 2). To confirm a distemper diagnosis, one or more of the following tests were performed on each animal: Complete blood count (CBC), rapid diagnostic test kit (Anigen Rapid CDV Ag Test Kit, BioNote, Hwaseong, Korea) from conjunctiva and/or cerebrospinal fluid (CSF) or RT‐PCR tests from whole blood and/or CSF. A definitive diagnosis of CDV infection was established if a positive result was obtained on any of the following tests: rapid test kit from conjunctiva or CSF, or RT‐PCR from blood or CSF. Additionally, efforts were made to differentiate distemper from other potential causes of neurological signs, such as parvovirus infection, food poisoning, and organophosphate poisoning. This differentiation process involved conducting parvovirus antigen tests on faecal samples to rule out parvovirus infection. Thorough clinical examinations and detailed patient histories were obtained to identify any potential exposure to toxins or contaminated food. Furthermore, we evaluated the progression and nature of clinical signs, as CDV typically presents with characteristic clinical signs such as myoclonus, which are less common in other conditions. These comprehensive diagnostic steps helped ensure that the neurological signs observed were indeed due to CDV infection rather than other potential causes.

**TABLE 1:** Characteristics, diagnostic test results, and outcomes of dogs in the distemper positive group.

Dogs No	Sex	Age (m)	Breed	Weight	Last vaccination	RKT_c_	RKT_csf_	RT‐PCR on blood	RT‐PCR on CSF	Clinical signs	Duration (Days)	Outcome
1	M	48	Terrier	5.6	No	—	+	—	—	Systemic, respiratory, increased reflexes (patellar and cranial tibia), LMN forelimbs	210	MR
2	F	8	GSD	20	No	—	—	—	+	Myoclonus (all limbs)	586	MR
3	M	6	Kuchi	25	No	+	Unk	+	Unk	Systemic, Stuporous, Seizure, Multiple neurologic signs	10	D
4	M	3	Mixed	4	No	+	Unk	+	Unk	Myoclonus (all limbs, diaphragm), Seizure, delayed hopping and knuckling (left forelimb and hind limb)	17	D
5	M	3	Mixed	8.2	No	+	+	+	+	Systemic, focal seizure (maxilla), increased reflex (cranial tibia, right hind limb)	7	D
6	F	3	Mixed	13	No	+	+	+	—	Systemic, Respiratory, Myoclonus (Chewing gum, diaphragm)	6	D
7	F	5	Mixed	13	No	Unk	+	+	+	Paresis (hind limbs), decreased reflex (cranial tibia)	17	D
8	M	6.5	Sarabi	34	No	+	+	+	+	Systemic, Respiratory, Ataxia, Myoclonus (left limbs, diaphragm)	10	D
9	F	18	Mixed	25	No	+	Unk	Unk	Unk	Systemic, Seizure, Myoclonus (different muscles of the body), Tetraplegia	5	D
10	M	Unk	Kuchi	17	No	Unk	—	+	+	Systemic Myoclonus (neck, forelimbs)	120	D
11	M	42	Kuchi	59	No	Unk	Unk	+	Unk	Myoclonus (left hind limb)	205	U
12	M	9	Terrier	6.6	No	+	Unk	Unk	Unk	Systemic Myoclonus (hind limbs, right forelimb)	150	E
13	F	7	Mixed	Unk	Unk	+	+	+	+	Systemic Myoclonus (hind limbs, jaw = chewing gum)	2	D
14	F	5	Mixed	6.3	No	Unk	+	Unk	Unk	Myoclonus (head, neck, right forelimb, diaphragm), Delayed postural reactions (right limbs)	Unk	Unk
15	F	2.5	Mixed	3.75	No	+	Unk	Unk	Unk	Systemic, Seizure, Myoclonus (head, neck, diaphragm, forelimbs, hind limbs), Delayed postural reaction (hind limbs)	2	D
16	M	48	Pointer‐setter	25	24 months ago	+	Unk	Unk	Unk	Myoclonus (head), Tetraplegia	34	D
17	M	48	Kuchi	45	24 months ago	+	Unk	Unk	Unk	Systemic s, Respiratory, Seizure, Delayed knuckling (hind limbs)	6	PR
18	F	5	Malinois	8	No	+	Unk	Unk	Unk	Systemic, Respiratory, Seizure, Delayed knuckling (right forelimb and hind limbs)	5	D
19	F	8	Mixed	19	No	+	Unk	Unk	Unk	Systemic Myoclonus (head)	7	D
20	M	5	Mixed	21	No	+	Unk	+	Unk	Systemic, Paresis (hind limbs)		D
21	F	9	Husky	17	No	+	Unk	Unk	Unk	Systemic Myoclonus (forelimbs, hind limbs)	60	D
22	F	4	Mixed	10	25 days ago	Unk	Unk	+	Unk	Myoclonus (hind limbs), decreased reflexes (all spinal reflexes), and delayed postural reactions	50	E
23	F	12	Mixed	33	No	+	Unk	+	Unk	Systemic, ataxia, myoclonus (forelimbs, abdominal muscles), delayed postural reaction (hind limbs)	3	E
24	F	12	Cavalier King Charles	3	180 days ago	+	Unk	Unk	Unk	Systemic, Ataxia		Unk
25	M	12	Spitz‐Terrier	Unk	No	Unk	Unk	+	Unk	Systemic, Myoclonus (right forelimb, diaphragm), Paresis (forelimbs), paralysis (hind limbs), Delayed postural reaction (all limbs)		Unk

Abbreviations: D, died; E, euthanised; MR, mostly recovered; PR, partially recovered; RKT_c_, rapid kit test on conjunctiva; RKTcsf, rapid kit test on CSF; U, unchanged; Unk, unknown.

**TABLE 2:** Characteristics, diagnostic test results, and outcomes of dogs in the distemper negative group.

Dogs No	Sex	Age (m)	Breed	Weight	Last vaccination	RKT_c_	RKT_csf_	RT‐PCR on blood	RT‐PCR on CSF	Clinical signs	Duration (Days)	Outcome
1	F	4	Mixed	Unk	+	—	—	—	—	Myoclonus (neck, shoulder, diaphragm, right hind limb)	400	U
2	F	6	Mixed	16	No	Unk	Unk	Unk	Unk	Myoclonus (neck, jaw = chewing gum), Seizure, Tetra paresis	30	D
3	M	3.5	Shih Tzu‐terrier	1.35	+	—	Unk	Unk	Unk	Myoclonus (periocular, neck, forelimbs, right hind limb), Seizure, Vertical nystagmus, Delayed hopping (all limbs), Increased reflexes (hind limbs)	Unk	Unk
4	M	60	Sarabi	50	No	Unk	Unk	Unk	Unk	Myoclonus (whole body), Tetraplegia, Seizure	5	D
5	M	3	Mixed breed of poodle	9	No	Unk	Unk	Unk	Unk	Seizure, Paresis, Delayed postural reaction (all limbs)	14	D
6	M	12	Mixed	12.5	Unk	Unk	Unk	Unk	Unk	Myoclonus (jaw = chewing gum, diaphragm, neck, right forelimb, left hind limb), Paraplegia, Delayed knuckling (hind limbs)	10	D
7	M	14	Shih Tzu‐Terrier	7.5	+	Unk	Unk	Unk	Unk	Systemic Myoclonus (jaw = chewing gum, neck), decreased reflexes (patellar)	30	D
8	F	5.5	Mixed	12	+	Unk	Unk	Unk	Unk	Systemic, Seizure, Myoclonus (diaphragm, left hind limb), Delayed knuckling (right forelimb and hind limb)	12	D
9	M	48	Great Dane	Unk	No	Unk	Unk	Unk	Unk	Myoclonus (head, right hind limb), decreased reflexes (patellar, cranial tibial)	2	D
10	M	22	Sarabi	60	Unk	Unk	Unk	Unk	Unk	Myoclonus (head)	30	D

Abbreviations: D, died; E, euthanised; MR, mostly recovered; R, recovered; RKT_c_, rapid kit test on conjunctiva; RKT_csf_, rapid kit test on CSF; U, unchanged; Unk, unknown.

### 2.1 Cerebrospinal Fluid Collection

Cerebrospinal fluid collection has been described elsewhere (Sarchahi et al. 2022b). Briefly, following a 12‐h food restriction, a cephalic vein catheter was placed for blood sampling (using EDTA tubes) and Ringer's solution administration (10 mL/kg/h). Anaesthesia was induced with an intravenous (IV) combination of ketamine (10 mg/kg) and diazepam (1 mg/kg). The atlantooccipital region (between the wings of the atlas and the occipital bone) was shaved and disinfected with Betadine followed by 70% alcohol. A sterile hypodermic needle was used to collect 0.5 mL of CSF per 10 kg of body weight. After collection, the animal was allowed to recover from anaesthesia. Distemper antigen presence was immediately evaluated using a rapid test kit on a portion of the CSF. The remaining CSF was frozen at ‐80°C for future analysis.

### 2.2 RT‐PCR Procedures

The detailed protocol for performing RT‐PCR analysis has been described elsewhere (Sarchahi et al. 2022a). Briefly, RNA was efficiently extracted from whole blood and cerebrospinal fluid (CSF) samples using commercially available kits following the manufacturer's instructions. cDNA was synthesised from the extracted RNA according to the manufacturer's recommended procedures. PCR amplification was carried out, targeting the CDV RNA as well as a housekeeping gene (GAPDH) as a control. The resulting PCR products were of the expected sizes. Sequencing of the PCR products confirmed the presence of CDV RNA. Sequence analysis showed a high degree of similarity to known CDV sequences in the GenBank database. The sequencing results showed the highest nucleotide similarity to canine distemper virus strain HL N (GenBank accession number EU489475.1) and canine morbillivirus strain PT61/Pt 2004 (GenBank accession number KX774415.1). This high level of sequence homology validates the specificity of the assay. The sequences identified in this study have been registered at GenBank with the following accession numbers: MZ707910 for PP‐I, MZ798146 for PP‐II, and MZ802994S for PP‐III.

### 2.3 Treatment Protocol

Dogs diagnosed with neurological distemper (excluding systemic, gastrointestinal and respiratory signs) received oral sodium phenobarbital (2.5 mg/kg) and prednisolone (0.55 mg/kg) every 12 h. The prednisolone dosage was tapered by half after 2 weeks and further reduced to a quarter after 1 month. Animals presenting with both neurological and systemic signs received the aforementioned medications alongside appropriate supportive care and antibiotics, as needed. The treatment was continued until clinical resolution of neurological signs or for a maximum of 3 months, whichever came first. Clinical resolution was defined as the absence of neurological signs for at least two consecutive weeks. The recovery or disease progression in each dog was monitored via weekly telephone consultations.

## 3 Results

Out of 35 dogs with obvious neurological signs related to CDV, 25 dogs had positive diagnostic test results, while ten dogs had negative results. The most important characteristics of dogs in the present study are listed in Tables 1 and 2. These tables present comprehensive data on the dogs in this study, including demographic information (age, sex, breed, weight, and vaccination status), diagnostic methods, neurological signs, duration of the disease and treatment, and outcomes. The average age of the dogs was 13.7 ± 15.4 months (range: 2.5–48 months), with an average weight of 18.3 ± 14.2 kg (ranging from 3 to 59 kg) in the positive test group. In this group, there were 12 male and 13 female dogs. Out of the twenty‐five dogs, 20 (80%) had no prior vaccinations, four (16%) were vaccinated (three of these dogs were over a year old and had previously received full vaccinations, but one of them was only 4 months old and had not yet been fully vaccinated, but its last vaccination was 25 days ago), and the vaccination status of one dog remains unknown. The predominant clinical sign observed in the dogs diagnosed with the neurological form of distemper (17 out of 25 dogs) was myoclonus (Table 1). Regarding treatment outcomes, among the 25 dogs in the positive group, two dogs (8%) were considered mostly recovered, one dog was partially recovered, one dog remained unchanged after 205 days of follow‐up, 18 dogs did not recover and died (15 naturally and three euthanised at the owner's request), and the fate of three remained undetermined due to the lack of owner response. To clarify, in our study, ‘mostly recovered’ refers to dogs that demonstrated significant improvement in mobility and overall function after treatment while still experiencing some residual deficits. For example, dog number 1 was completely paralysed in its hind legs before treatment and could not bear weight; however, it was able to walk independently 1.5 months after starting treatment. Initially, it staggered but eventually walked with only occasional falls and some dragging of its legs. Dog number 2 had myoclonus in all four limbs before treatment, with myoclonus being mild in three limbs and severe in one limb. After treatment, there was a significant reduction in myoclonus in the weaker legs, but the leg that previously exhibited severe myoclonus continued to show lameness, causing the animal to walk normally on three limbs while having a lameness in one limb. In contrast, ‘partially recovered’ pertains to dogs that showed some improvement but did not achieve full functionality. Dog number 17 had both systemic symptoms and neurological signs, including seizures. While the systemic symptoms and seizures resolved, the owner reported only partial recovery due to ongoing neurological deficits. It is important to note that the assessment of dog number 17 was based on the owner's statements and may not reflect an accurate clinical evaluation; therefore, it was not classified as a recovered patient in our study, but dogs 1 and 2 were carefully examined by us, and our descriptions of their neurological status are based on direct clinical observations. In the group with obvious CD neurological signs but negative test results, eight out of ten dogs died, one dog's condition remained unchanged after 400 days of follow‐up, and the outcome of the remaining case was uncertain (Table 2).

Of the 25 dogs in the positive group, only seven dogs (28%) presented with seizures in addition to other symptoms. Among these seven dogs, four (57%) remained seizure‐free during treatment until death or recovery. However, three dogs (43%) continued to experience seizures despite anticonvulsant treatment. Notably, one dog that did not initially present with seizures developed seizures at the time of death, despite receiving anticonvulsant treatment. This indicates that while anticonvulsant therapy was effective in managing seizures for the majority of affected dogs, it was not universally successful, and seizures could still develop as a late‐stage complication in some cases.

In the positive group, out of 25 patients, the duration of the disease was unknown in 5 patients. Among the remaining 20 cases, the time from referral to the clinic until the end of follow‐up ranged from 2 to 586 days, with an average of 72 ± 135.2 days. The median follow‐up time was 10 days (interquartile range: 5.5–90 days). Regarding the negative group, out of 10 cases, the duration of the disease was unknown in one case. In 9 cases, the disease persisted for 2 to 400 days, averaging 59.2 ± 128.3 days (*p* = 0.91). The median follow‐up time was 14 days (interquartile range: 7.5‐30 days). In the positive group, seven dogs (28%) exhibited solely neurological signs, while 18 (72%) displayed both neurological and systemic signs, with similar mortality rates in both subsets. Notably, one dog that mostly recovered presented only neurological signs, while another exhibited both neurological and systemic signs. These dogs were monitored for 210 and 586 days, respectively, with no reported complications. In the current study, four out of the 25 infected dogs had received prior vaccinations (16%). Of these four vaccinated dogs, two dogs manifested solely neurological signs. Unfortunately, these two dogs either died or were euthanised. One dog exhibited both neurological and systemic signs but partially recovered, and the outcome for the fourth dog remains unclear.

## 4 Discussion

Canine distemper is a severe and often fatal disease, with a particularly poor prognosis for recovery, especially in its neurological form (Ranjithkumar and Dey 2021; Sarchahi et al. 2022b). Despite ongoing research efforts, a safe and effective method to significantly improve the recovery rate from this disease has yet to be identified. Scientists have long been searching for ways to enhance the recovery rate of canine distemper. Various treatment approaches have been studied, but none have reported a high rate of successful recovery (Teixeira et al. 2009; Bogdanchikova et al. 2016; Liu et al. 2016; Arbabi et al. 2022; Sarchahi et al. 2022b).

The canine distemper virus gains access to the central nervous system primarily through infected immune cells, particularly monocytes expressing the signalling lymphocytic activation molecule (SLAM). These infected cells carry the virus across the blood‐brain barrier. Once in the CNS, the virus causes lesions through two mechanisms. First, direct viral effects, including the infection of neurones, astrocytes, and olfactory ensheathing cells, lead to neuronal necrosis, eosinophilic inclusion body formation, and neuronophagia. Second, immune system activation contributes to immunopathology, characterised by demyelination, progressive perivascular mononuclear infiltration, astrogliosis, and gliosis. These lesions vary in severity and are influenced by the virus strain, host immune response, and phase of infection. The acute phase often involves spongy vacuolation, endothelial swelling, and reactive gliosis, while chronic lesions show extensive demyelination and inflammatory cell infiltration (Beineke et al. 2009; Rudd et al. 2010; Zhao and Ren 2022).

Therefore, in some newly infected patients, the virus is detectable in the central nervous system, whereas in chronic cases, only residual effects from the virus (both direct viral lesions and secondary immunological effects) persist without detectable virus presence in nervous tissues (Beineke et al. 2009; Sarchahi et al. 2022a). The use of corticosteroids has been proposed for treating the neurological form of distemper; however, scientific evidence supporting their efficacy remains lacking (Sykes 2022). Ranjithkumar and Dey have reported that corticosteroids do not offer any beneficial effects in treating the neurological form of distemper (Ranjithkumar and Dey 2021). Their study, which included 47 serologically confirmed cases of neurologic canine distemper, revealed that glucocorticoid usage, such as betamethasone, failed to prevent the progression of disease from systemic to neurologic forms or vice versa. Among the seven cases treated with corticosteroids, no clinical improvement was observed. This finding underscores their conclusion that glucocorticoid therapy does not confer any clinical advantage in managing neurologic distemper cases. In the current study, only two dogs exhibited significant recovery, while many dogs with neurological distemper died shortly after initiating treatment or were euthanised due to ineffective treatment upon the owner's request. This suggests that treatment efficacy is largely dependent on the initial condition of the animal. Animals in poor condition at the onset of treatment typically do not respond well and experience a rapid decline, whereas those with neurological signs but overall good health and appetite tend to survive longer, with treatments showing greater efficacy in such cases. However, we acknowledge that our ability to assess treatment effectiveness in severely affected animals was limited; those with very severe initial conditions did not survive long enough for us to evaluate the impact of the treatments adequately. This limitation highlights a critical gap in our understanding and suggests that further research is necessary. While we cannot ethically conduct additional studies on severely affected patients due to their rapid decline, focusing on animals with better initial health and appetite provides valuable insights into treatment efficacy. The two cases where significant recovery was observed support our hypothesis that initial condition plays a crucial role in treatment outcomes.

In the present study involving 35 dogs, a small proportion of the dogs exhibited seizures following the initiation of treatment. Notably, seizures are a hallmark clinical sign of canine distemper with neurological involvement (Ranjithkumar and Dey 2021). Our findings suggest a lower incidence of seizures compared to some previous reports, which may indicate a potential benefit of the treatment protocol used in our study. However, given our small sample size and the lack of a control group, further research is needed to confirm whether this reduction in seizure occurrence is directly attributable to the management approach. While our results are encouraging, larger controlled studies are necessary to determine if dogs diagnosed with canine distemper could potentially lead complication‐free lives for extended periods with the aid of anticonvulsant therapy. Conversely, in our study, drugs were shown to have limited effectiveness in dogs presenting with evident paralysis, leading to a rapid decline and mortality. Dogs displaying only myoclonus, however, may have a prolonged lifespan with this singular sign. Notably, the efficacy of prednisolone in these dogs was limited, with only 8% (2 out of 25) experiencing recovery, reflecting a low success rate in our study.

One limitation of the present study is the relatively small sample size of patients examined. While a larger sample size typically yields more robust results, this study faced several practical constraints, such as the financial burden on animal owners, challenges in animal maintenance, and the necessity for conducting multiple diagnostic tests. Consequently, a significant number of patients were excluded during the study.

Another limitation is the lack of cooperation from numerous dog owners regarding sampling and treatment, leading to uncertain outcomes and a reduction in the study's patient population. The results show a low response rate to corticosteroid treatment, with only two cases (8%) showing improvement. Although these cases presented relatively severe clinical signs, they were not severe enough to result in rapid death. This low response rate suggests limited efficacy of corticosteroids in our study population. Regarding seizure management in canine distemper, our findings reflect the complex nature of this aspect of the disease. The effectiveness of phenobarbital in managing seizures varies among individual cases. While phenobarbital can be effective for many dogs, it is not universally successful. Some dogs may require additional medications or higher doses to achieve adequate seizure control. The development of seizures as a late‐stage complication, even in dogs initially responding well to treatment, highlights the progressive nature of canine distemper and the challenges in its long‐term management. This underscores the variability in individual cases and the need for tailored treatment approaches in managing seizures associated with canine distemper.

## 5 Conclusions

Our study provides insights into the prognosis of canine distemper with neurological signs, though the small sample size limits the strength of our conclusions. We observed that the majority of dogs (18 out of 25) in the positive group with severe initial illness did not recover, suggesting that disease severity at presentation may influence prognosis. In our limited sample, four out of seven dogs presenting with seizures responded to anticonvulsant therapy, indicating potential for management in some cases. However, the overall low recovery rate (8%) in our study, particularly in dogs with multiple neurological manifestations, suggests that current treatment modalities, including corticosteroids, may have limited efficacy in severe cases. Given these observations and the limitations of our study, further investigations with larger sample sizes and controlled designs are warranted to evaluate the efficacy of current and novel therapeutic approaches for canine distemper with neurological involvement.

## Author Contributions

Conceptualisation: Ali Asghar Sarchahi. Methodology: Ali Asghar Sarchahi, Mohammad Arbabi, Hadi Mohebalian. Writing – original draft preparation: Ali Asghar Sarchahi, Mohammad Arbabi. Writing – review and editing: Ali Asghar Sarchahi, Mohammad Arbabi, Hadi Mohebalian. Supervision: Ali Asghar Sarchahi

## Ethics Statement

This study was conducted in accordance with the guidelines for the care and use of animals in research and was approved by the ethics committee of Ferdowsi University of Mashhad, Iran (https://ethics.research.ac.ir/IR.UM.REC.1399.120).

## Consent

The treatment protocol was explained to the animal's owners and was started with their consent. The authors have nothing to report on consent to publish.

## Conflicts of Interest

The authors declare no conflicts of interest.

## Peer Review

The peer review history for this article is available at https://publons.com/publon/10.1002/vms3.70479.

## Data Availability

All data generated or analysed during this study are included in this published article. The datasets used and analysed in this study are available from the corresponding author on reasonable request.
